# Medical treatment of Attention Deficit/Hyperactivity Disorder (ADHD) and children’s academic performance

**DOI:** 10.1371/journal.pone.0207905

**Published:** 2018-11-29

**Authors:** Maria Keilow, Anders Holm, Peter Fallesen

**Affiliations:** 1 Department of Education, VIVE–The Danish Center for Social Science Research, Copenhagen, Denmark; 2 Mindhood–Child Mental Health Research Program, Department of Public Health, Aarhus University, Aarhus, Denmark; 3 Department of Sociology, Western University, London, Canada; 4 The ROCKWOOL Foundation, Copenhagen, Denmark; 5 The Swedish Institute for Social Research, Stockholm University, Stockholm, Sweden; Harvard Medical School, UNITED STATES

## Abstract

Attention Deficit/Hyperactivity Disorder (ADHD) is negatively associated with a range of academic achievement measures. We use Danish administrative register data to study the impact of medical treatment of ADHD on children’s academic performance assessed by student grade point average (GPA). Using administrative register data on children, who begin medical treatment, we conduct a natural experiment and exploit plausible exogenous variation in medical nonresponse to estimate the effect of medical treatment on school-leaving GPA. We find significant effects of treatment on both exam and teacher evaluated GPAs: Compared to consistent treatment, part or full discontinuation of treatment has large significant negative effects reducing teacher evaluation and exam GPA with .18 and .22 standard deviations, respectively. The results demonstrate that medical treatment may mitigate the negative social consequences of ADHD. Placebo regressions indicate that a causal interpretation of our findings is plausible.

## Introduction

One in five individuals experiences the onset of mental health problems before reaching adulthood [1]. Mental health problems in childhood have substantial consequences for later life because such problems are associated with lower educational achievement and attainment. Attention Deficit/Hyperactivity Disorder (ADHD) represents one such mental health problem, which infers substantial individual and societal costs if left untreated [2]. Children with ADHD have lower grade point average (GPA), do worse on academic tests, have higher retention rates and absenteeism, and have lower high school and college completion rates [3–9].

Recent research has linked ADHD to delayed maturation of parts of the brain directly related to cognition [10–12], indicating a direct biological link between ADHD and low educational achievement. In addition, ADHD may also negatively affect the learning environment in children’s homes. An emerging body of work demonstrates that undiagnosed or untreated ADHD leads to increased family instability, such as increased risk of parental union dissolution [13–14] and for entering an out-of-home placement [15]. Thus, beyond the direct effect on cognitive abilities, ADHD may cause lower educational achievement and attainment through affecting life circumstances outside the classroom, leaving affected children double disadvantaged.

One of the modal ways to address the behavioral problems related to ADHD is through medical treatment with psychotropic drugs. Although the use of these drugs is not uncontroversial [16–19], drug therapy has been consistently shown to address core symptoms among children with moderate to severe ADHD [20]. If ADHD affects educational outcomes negatively, successful treatment could reduce the negative educational consequences caused by ADHD.

This study tests whether efficient medical treatment of ADHD affects children’s academic performance by utilizing a natural experiment. The medical literature documents that 25 to 30 percent of treated individuals with ADHD are *nonresponders*. Nonresponders are unaffected by the medication or have severe side effects, in either case causing them to discontinue treatment even though they show the same symptoms as responders prior to treatment [21]. There do not appear to be significant socioeconomic differences between responders and nonresponders [22–23] nor do individual characteristics (age, gender, IQ, ADHD subtypes and symptoms, neuropsychological characteristics) predict treatment response [24–25]. Under this assumption of no significant differences between responders and nonresponders, we compare the academic performance of children diagnosed with and treated for ADHD, who either discontinue treatment (nonresponders) or continue treatment (responders) to estimate the effect of medical treatment on academic performance. In our study, we have no selection into treatment as all individuals in our analysis initiate treatment. We may have selection out of treatment. However, as argued above, we believe that selection out of treatment is random. We substantiate this below.

We find that medical treatment has a significant and sizeable effect on long-term academic performance outcomes in terms of school-leaving GPA. The effect of medical treatment of ADHD is comparable in size to the gender differences in GPA. To test whether a causal interpretation of the results is plausible and to account for any social gradient in treatment patterns, we estimate the effect of treatment with and without a rich set of potential confounders and perform placebo regressions on a sample of children, who initiate medical treatment *after* graduation (i.e. after receiving their final grades). We find no significant differences in the effect of treatment with and without confounders and we find no differences in the GPA between medical responders and nonresponders in the placebo sample, indicating that the main results likely express causal effects. Our results demonstrate that medical treatment of ADHD may play a substantial role in diminishing educational disparities caused by the condition.

The remainder of this paper is organized as follows: Section 2 reviews relevant literature and theories on the effect of ADHD and ADHD treatment on educational outcomes. Section 3 presents the data for our empirical analysis. Section 4 presents our identification strategies and statistical method. Section 5 presents results. Section 6 discusses the findings and concludes.

## ADHD and educational outcomes

Untreated behavioral problems may substantially impair children’s learning and educational achievement [26–27]. Previous studies have found that children diagnosed with ADHD on average attain 2.2 to 2.5 years less schooling than non-ADHD peers do, and 25 percent of students with ADHD drop out of high school [5,8,9,27]. Recent work has further shown that both externalizing behaviors and attention deficits contribute to the lower educational attainment [7]. Children with ADHD also display lower cognitive achievements, lower test scores, and higher scholastic impairment [5,28], with especially attention problems predicting poorer math and reading achievement [29–30]. Thus, research consistently shows that ADHD affects a number of various educational outcomes ranging from performance and achievement measures to school behaviors. In this study, we focus specifically on student academic performance assessed by teacher evaluation and exam GPA. In the remainder of this section, we discuss biological and social mechanisms linking ADHD to decreased educational achievement outcomes, as well as how efficient medical treatment can sever parts of the link.

### Double disadvantage: Biological and social consequences of ADHD and educational outcomes

The research literature on the consequences of ADHD for children’s lives indicates two mutually reinforcing pathways through which the disorder affects educational outcomes: direct biological effects on cognitive development and indirect effects on children’s home environment that likely affect learning.

Neurological research has shown that children with ADHD experience delayed maturation of the brain—more specifically delayed growth of the pre-frontal cortex, i.e. the part of the brain associated with the regulation of executive functions, such as complex cognition, decision-making processes, and the moderation of social behavior. In their seminal longitudinal study of 223 children diagnosed with ADHD and 223 typically developing controls, Shaw et al. [10] show substantial delay in the maturation of the pre-frontal cortex among children with ADHD. Among children with ADHD, 50 percent of cortical points had reached peak thickness at age 10.5 years, three years later than among the typically developed controls. More recent work by Sripada, Kessler and Angstadt [11] further renders probable that children with ADHD also experience delayed maturation of deeper parts of the brain’s architecture and pathways, which are associated with attention, control of impulsivity, disregard of irrelevant stimuli, and other cognitive tasks. In total, there is strong evidence that delayed neurobiological development of areas of the brain that moderate behavior is important for conductive learning-behavior among children with ADHD. The neurobiological findings dovetail with studies of educational performance among children with ADHD, which find that both inattention and externalizing behavior predicts worse educational outcomes [5,7,28–30].

Yet, delayed cognitive maturation may not be the only cause driving the relationship between ADHD and low educational achievement. Neither learning nor ADHD occur in a social vacuum. Beyond the direct neurobiological effects, ADHD also places strain on children’s life circumstances outside the educational system in ways that could deter learning. Untreated ADHD increases the risk of experiencing family instability, such as parental divorce [13–14] and, more dramatically, the risk of entering an out-of-home placement [15]. The association between family instability and lower educational achievement is well-established, although recent work has called (the generalizability of) causal claims into question [31–33]. Yet, for children with ADHD, family stability could likely worsen an already vulnerable learning situation. Thus, the effect of ADHD on educational achievement is likely not only driven directly by delayed maturation of the brain, but also by secondary effects of ADHD on children’s life and learning circumstances. Because research has shown that medical treatment of ADHD both mitigates the direct behavioral symptoms and lowers the risk of family instability and engaging in risky behavior, treatment could also have substantial impact on educational achievement.

### Medical treatment of ADHD and educational outcomes

The strong association between ADHD and the above-mentioned host of negative outcomes has given rise to a number of treatment types, with pharmacological treatment with stimulants being the most common form [20,34]. Overall the short term efficacy of medical treatment on core ADHD-symptoms is well documented for the age group 6–18 years by over 200 clinical RCT’s since the 1960s [20]. Short-term medical treatment effects on academic achievement outcomes have also been studied extensively and show positive effects [35], whereas long-term studies of the effects on education are scarce and effects are generally more diverse, smaller, or less significant than short-term effects [36]. In their recent review, Baweja et al. [3] conclude that the evidence for a positive effect of medical ADHD treatment on school performance remains more substantial for acute than long-term indicators of academic performance. Baweja et al. [3] also note that evidence is strongest for the effect of stimulants to improve teacher ratings of behavioral outcomes. Methodological problems, such as sample attrition, are a probable explanation for the lack of empirical support for long-term effects, wherefore the want of research on this matter is substantial [35]. Nevertheless, as outlined above, the substantial number of both clinical and non-clinical findings on the impact of medical treatment of ADHD on domains of children’s lives connected with education do suggest that medical treatment could increase educational achievement among affected children.

## Data

### Data sources

We use data from Danish administrative registers collected by Statistics Denmark. The primary data sources are the Danish Registry of Medicinal Product Statistics (RMPS) and the national registers on educational attainment (REA). Danish registers offer extensive individual-level information on all educational courses commenced in the public educational system and various academic performance measures. Register data has almost no sample drop out or attrition and validity and coverage is very high; e.g. in 2008, 96 percent of the Danish population aged 15–69 have non-missing education information [37]. From 2002, REA contains teacher evaluation grades and exam grades of students, who graduate the compulsory level of schooling (ninth grade in Denmark). From these school-leaving grades, we create our outcome measures by calculating standardized overall teacher evaluation and exam GPA.

Data from RMPS contain detailed information on all legal purchases of prescription drugs in Denmark (date, type of drug, dosage, etc.). The prescription data are linked to the individual identification number of the patient through the Danish health insurance system [38]. By using register data we avoid bias from refusal of participation, which is often a problem for survey data studies of ADHD [39–40] as well as recall bias from self-reported information on medical treatment patterns and academic performance. The RMPS is found to have a high completeness and validity, which is mainly due to reimbursement-driven record-keeping with automated bar-code-based data [40–41].

### Operationalization of treatment patterns

In order to determine the effect of medical treatment of ADHD, we use longitudinal data from RMPS and define three types of pharmacological treatment patterns of use in the analysis: *Discontinued Pharmacological Treatment* (DPT), *Ambiguous Pharmacological Treatment* (APT), and *Continuous Pharmacological Treatment* (CPT). The difference between these three types of treatment allows us to estimate the effect of treatment by comparing the outcome across the three different treatment patterns with the DPT acting as a control group and the CPT as a treatment group and the APT as a partial compliance group. The definition of discontinuation (DPT) follows strict restriction rules: we define DPT as having purchased medication for maximum three months within the data window, which just allows for initial medicine trial and dose titration. Likewise, the definition of continuous treatment (CPT) is restricted to those, who have a regular and stable use of medication with purchases being no more than three months apart. The patterns of the remaining group, the APTs, are then per definition ambiguous as they cannot be convincingly placed in either the DPT or CPT category. These are children, who repeatedly enter in and out of medical treatment. All three categories are mutually exclusive. In all analyses, CPT is reference group, whereby we investigate inverse treatment effects of DPT and APT (discontinuation and ambiguous treatment) compared to CPT (continuous treatment).

### Sample criteria

We sample all children diagnosed with and treated for ADHD from the total population of children, who completed Danish compulsory schooling from 2002 to 2011. To identify medically treated children (irrespective of their subsequent treatment pattern), we select children, who redeem at least one prescription for any type of ADHD medication (methylphenidate, atomoxetine, or modafinil) between age twelve and prior to school-leaving exams (*treatment sample)*. Note that amphetamine salts were not introduced in Denmark for treatment of ADHD until 2013 due to official national restrictions and that these types of drugs are therefore not included in our sample criteria. We exclude children, who begin treatment before age twelve because scholastic impairment is likely less traceable among children at this age and because they more likely suffer from hyperactivity than attention problems (the latter of which especially predict lower academic achievement). All analyses were also conducted for the total sample yielding very similar results. We define an additional sample of children, who start treatment after school-leaving exams and use this as a *placebo sample* (we elaborate on this in section 4 on methods).

We restrict both samples further using the following criteria. First, children who either discontinue medical treatment within the first three months of the RMPS data window or begin treatment during the last three months of the data window are excluded from analyses, as the treatment patterns of these children cannot be convincingly determined. Second, the sample is restricted to cases with valid information on main outcome variables; teacher evaluation and exam GPA. In our data, 92 percent of all children have valid information for GPA, compared to 70 percent of children diagnosed with and treated for ADHD. For students in public schools, the school-leaving examinations are obligatory, but exemptions can be applied for (reasons for exemption could be severe functional impairments) [42]. Exams are not obligatory in private schools, though a school-leaving diploma is required for almost all upper secondary educational tracks.

Third, we exclude children, who commence treatment around the time point of exams to minimize the risk that we include children, whose (expected) poor exam performance causes them to commence treatment to boost future academic performance [43]. These restrictions result in a treatment sample consisting of 2659 individuals from the birth cohorts 1984–1996, and, correspondingly, a placebo sample of 3785 individuals from the birth cohorts 1983–1994.

### Variables

To account for any effect of the duration of medical ADHD treatment, severity of symptoms, or potential correlations between age and treatment response [36], we control for individual age at treatment start. Age at graduation is included to control for grade retention. We further control for the medical dosage using the number of Defined Daily Doses (DDD) [44] for the individuals’ first and third purchase of ADHD medication. The severity of ADHD symptoms (which is unobserved) may both influence dosage and whether an individual child continues treatment [24–25]. Controlling for dosage titration may break the correlation between severity of the condition and treatment continuation. Hence, if controlling for medication dosage alters the estimated effect of treatment continuation this is evidence that severity and continuation are correlated. To control for comorbidity issues [45], we also include information on use of psychoactive medication for conditions other than ADHD. Data on any comorbid diagnoses are not, however, available.

To account for the event that treatment patterns (DPT and APT versus CPT) vary across child and family characteristics, we include a rich set of control variables. Individual level information includes gender, immigrant status, birth weight, and gestational age. Studies show that birth characteristics predict ADHD symptoms [46–47] and correlate with academic performance measures such as GPA [48]. We also include a dummy indicator for whether or not the child was graded using a new Danish grading scale introduced in 2008 [49] to account for any impact of this change on our results. (Grades from before 2008 are converted to the grade scale for 2008 using the official grade conversion table. The dummy variable thus captures any potential differences in grading across 2007/2008 that are not fully accounted for by the conversion of grades. The estimate of the dummy variable is insignificant in all analyses).

We measure parental characteristics one year prior to the birth of the child to avoid post treatment confounding. We control for parental income (including dummies for negative income generated by negative income from self-employment), unemployment, educational level (ISCED code dummies), parent age at the birth of their child, and whether either parent has an ADHD diagnosis.

Table 1 presents the descriptive statistics of all background variables. We show data for the population of the full population sample of 2002–2011 graduates and for our restricted treatment and placebo sample. Of the most notable differences, the proportion of males and non-immigrant Danes are higher in both the treatment and placebo sample compared to the population. The gender difference is most pronounced in the treatment sample, however. The overrepresentation of males and ethnic Danes is consistent with clinical accounts of diagnostic patterns for ADHD. Parents of children in the ADHD samples are slightly younger, more likely to have an ADHD diagnosis, have higher unemployment rates, lower income, and a greater proportion of parents have lower educational attainment levels. For several characteristics, there are notable differences between the treatment and placebo sample. However, as we show in the results section, these differences do not seem to alter how medical treatment affects the outcome. We offer explanations as to why below.

**Table 1:** Descriptive statistics for all background variables. Population sample of compulsory school graduates from years 2002–2011 and the ADHD sample.

	Population sample			ADHD sample					
				Treatment sample			Placebo sample		
	Mean	SD	N	Mean	SD	N	Mean	SD	N
Individual characteristics									
Male	0.50	0.50	571107	0.70*	0.46	2659	0.58*	0.49	3785
Gestational age	39.58	1.80	529033	39.39*	1.99	2494	39.52	1.88	3545
Birth weight	3460.78	567.00	531476	3461.95	60.49	2508	3386.56*	582.17	3562
Immigrant status:									
Non-immigrant (ref.)	0.91	0.29	571072	0.95*	0.21	2659	0.96*	0.20	3785
Immigrant	0.04	0.21	571072	0.02*	0.15	2659	0.03*	0.16	3785
Descendant of immigrant	0.05	0.22	571072	0.02*	0.15	2659	0.02*	0.13	3785
Age at graduation	16.13	0.46	571104	16.40*	0.65	2659	16.24	0.57	3785
Graded with the new rating scale (since 2008)	0.42	0.49	571107	0.77*	0.42	2659	0.08*	0.27	3785
Birth cohort^a^	1990.53	2.84	571107	1992.34*	2.29	2659	1988.54*	1.91	3785
Age at treatment start				14.57	1.54	2659	20.93	1.93	3785
ADHD treatment pattern:									
DPT				0.11	0.31	2659	0.32	0.47	3785
APT				0.70	0.46	2659	0.38	0.49	3785
CPT				0.19	0.39	2659	0.30	0.46	3785
Dosage, first purchase of ADHD medication^c^				14.37	10.87	2659	13.79	9.31	3785
Dosage, third purchase of ADHD medication^c^				18.56	13.12	2659	16.36	13.86	3785
Ever purchased any psychoactive medication	0.11	0.31	571107	0.16*	0.36	2659	0.45*	0.50	3785
Average purchases of psychoactive medication^d^	0.34	0.47	62273	0.14*	0.31	2659	0.10*	0.24	1685
Residential region at age three:									
Capital (ref.)	0.27	0.45	542025	0.32*	0.47	2565	0.23*	0.42	3666
Zealand	0.14	0.35	542025	0.14	0.35	2565	0.13	0.34	3666
South Denmark	0.23	0.42	542025	0.16*	0.36	2565	0.17*	0.38	3666
Central Jutland	0.24	0.42	542025	0.27*	0.44	2565	0.34*	0.48	3666
Northern Jutland	0.11	0.31	542025	0.10	0.31	2565	0.11	0.32	3666
Bornholm	0.01	0.09	542025	0.01	0.12	2565	0.01	0.10	3666
Residential region at graduation age:									
Capital (ref.)	0.25	0.43	570740	0.26	0.44	2659	0.19*	0.40	3784
Zealand	0.16	0.37	570740	0.17	0.38	2659	0.16	0.36	3784
South Denmark	0.23	0.42	570740	0.16*	0.37	2659	0.17*	0.37	3784
Central Jutland	0.24	0.42	570740	0.28*	0.45	2659	0.35*	0.48	3784
Northern Jutland	0.11	0.32	570740	0.11	0.31	2659	0.12	0.33	3784
Bornholm	0.01	0.11	570740	0.02	0.15	2659	0.01	0.10	3784
Parent characteristics									
Mother diagnosed with ADHD	0.01	0.07	571107	0.08*	0.28	2659	0.07*	0.25	3785
Father diagnosed with ADHD	0.00	0.06	571107	0.05*	0.22	2659	0.03*	0.18	3785
Mother’s age at childbirth	28.57	4.77	568662	28.13*	4.84	2655	27.19*	4.90	3779
Father’s age at childbirth	31.53	5.68	520182	30.99*	5.73	2308	30.40*	5.86	3324
Mother’s education^b^:									
No education (ref.)	0.14	0.35	571107	0.08*	0.27	2659	0.12*	0.32	3785
ISCED 2	0.17	0.38	571107	0.23*	0.42	2659	0.31*	0.46	3785
ISCED 3A	0.08	0.28	571107	0.10*	0.30	2659	0.07*	0.25	3785
ISCED 3C	0.38	0.48	571107	0.41*	0.49	2659	0.34*	0.47	3785
ISCED 5B	0.19	0.39	571107	0.15*	0.36	2659	0.13*	0.34	3785
ISCED 5AC	0.04	0.20	571107	0.03*	0.17	2659	0.02*	0.15	3785
ISCED 6	0.00	0.02	571107	NA	NA	2659	0.00	0.02	3785
Father’s education^b^:									
No education (ref.)	0.26	0.44	571107	0.25	0.43	2659	0.30*	0.46	3785
ISCED 2	0.11	0.32	571107	0.16*	0.37	2659	0.16*	0.37	3785
ISCED 3A	0.05	0.22	571107	0.04*	0.20	2659	0.04*	0.20	3785
ISCED 3C	0.40	0.49	571107	0.41	0.49	2659	0.37*	0.48	3785
ISCED 5B	0.11	0.31	571107	0.09*	0.29	2659	0.08*	0.27	3785
ISCED 5AC	0.07	0.25	571107	0.05*	0.22	2659	0.05*	0.21	3785
ISCED 6	0.00	0.05	571107	0.00	0.04	2659	0.00	0.04	3785
Mother’s yearly income (USD, 2009 prices)^b^	40309	21140	541964	38901*	18218	2659	36501*	16723	3688
Father’s yearly income (USD, 2009 prices)^b^	58432	53511	501487	47987*	32395	2659	54118*	36316	3264
Mother’s has negative income^b^	0.00	0.03	571107	NA	NA	2659	0.00	0.03	3785
Father has negative income^b^	0.00	0.05	571107	0.00	0.04	2659	0.00	0.04	3785
Mother’s unemployment rate^b^	138.85	253.76	535712	173.90*	276.52	2567	177.98*	272.42	3647
Father’s unemployment rate^b^	75.77	191.92	496443	98.24*	214.20	2268	103.14*	215.03	3231

*Source*: Own calculations on data from Statistics Denmark.

* indicates that the mean of the ADHD sample is significantly different from the population sample mean at the 5 percent level of signification (estimated from t-tests). NA denotes zero responses for the variable in question. Unweighted data. Treatment sample: Children who begin medical ADHD treatment *prior to* compulsory school graduation. Placebo sample: Children who begin medical ADHD treatment *after* compulsory school graduation.

^a^ The analyses use dummies for birth cohort.

^b^ Information on parent educational attainment, income, and unemployment is measured one year prior to the birth of the respondent in order to avoid post treatment confounding.

^c^ Medication dosage is measured in DDDs (Defined Daily Dosages).

^d^ Average purchases of psychoactive medication is calculated as average number of purchases of any type of psycholeptic or psychoanaleptic medication per month (excluding medicine for ADHD).

Table 2 presents descriptive statistics on outcome measures. Teacher evaluation GPA reflects students’ academic performance in class as evaluated by the teacher, whereas exam GPA reflects assessments made by the students’ teacher as well as an external examiner during the school-leaving exams (oral and written). Whereas teacher evaluation GPA thus reflects students’ behavior as well as their academic performance, exam GPA can be interpreted as an estimate of student academic performance. Because exams include an external assessment as well as written exams, student behavior that is unrelated to their actual achievement less likely influences these assessments. Grades are assessed using the Danish 7 point grading scale, which corresponds to the European Credit Transfer and Accumulation System (ECTS)-scale [49]. All empirical models use GPAs standardized within the population sample. Both the main ADHD sample and the placebo sample have substantially and significantly lower GPAs than found for the full population.

**Table 2:** Descriptive statistics for unstandardized and standardized dependent variables. Population sample of compulsory school graduates from years 2002–2011 and the ADHD sample.

	Population sample			ADHD sample					
				Treatment sample			Placebo sample		
	Mean	SD	N	Mean	SD	N	Mean	SD	N
Unstandardized variables									
Exam GPA	6.23	2.22	571107	4.81*	2.09	2659	4.71*	1.98	3785
Teacher Evaluation GPA	6.34	2.22	571107	4.72*	2.05	2659	4.64*	2.00	3785
Standardized variables									
Exam GPA	0.00	1.00	571107	-0.64*	0.94	2659	-0.69*	0.90	3785
Teacher Evaluation GPA	0.00	1.00	571107	0.73*	0.92	2659	-0.77*	0.90	3785

*Source*: Own calculations on data from Statistics Denmark.

* indicates that the mean of the ADHD sample is significantly different from the population sample mean at the 5 percent level of signification (estimated from t-tests). GPA: Grade point average. Unweighted data. Treatment sample: Children who begin medical ADHD treatment *prior to* compulsory school graduation. Placebo sample: Children who begin medical ADHD treatment *after* compulsory school graduation.

## Method

We estimate the effect of medical treatment on school-leaving GPAs for a sample of children diagnosed with ADHD, who initiate medical treatment. Our design has the advantage of circumventing selection into diagnosis and treatment of ADHD, as all children in the sample have been diagnosed and have initiated treatment. However, selection out of treatment may be an issue. As mentioned previously, we exploit evidence from medical research showing that individual response towards ADHD medication regarding the improvement of core symptoms is arbitrary with respect to individual characteristics of the patient [22–23,25,36]. Consequently, among a group of individuals diagnosed with ADHD, whether or not the medication reduces individual symptoms is from a medical perspective as good as random.

### Analytical setup

To measure the effect of medication on students’ school-leaving GPA we estimate the following regression:

$$GPA_{i}=X_{i}\beta +\gamma_{1}DPT_{i}+\gamma_{2}APT_{i}+e_{i}$$ (1)

where *GPA*_*i*_ is student GPA, *X*_*i*_ is a vector of individual characteristics, *β* is a vector of corresponding regression coefficients, DPT and APT are binary indicators of discontinued treatment and ambiguous treatment. *e*_*i*_ is the error term. The regression coefficient *γ*_*1*_ measures the average difference in outcomes between those continuing medication (CPT) and those discontinuing medication (DPT) from the onset of diagnosis and until completing compulsory school, where GPA is measured.

The interpretation of *γ*_*2*_ is less clear-cut, but captures the average difference in outcome between a continued treatment pattern and the *average* ambiguous treatment pattern (APT). The effect thus both depends on the effectiveness of the medication (generating a difference between those with an ambiguous and a continuous medical consumption) and it depends on the distribution of the ambiguous treatment patterns in the data. If APT patterns on average are close to full compliance, children in this group should have the same outcome as the CPTs. If, however, they, on average, are close to a full discontinuation of treatment, children in this group should, on average, have the same outcome as the DPTs. At any rate, a negative estimate of *γ*_*2*_ indicates that medication is effective.

Although random nonresponse is supported by empirical evidence, drop out from medical treatment in observed data may be nonrandom for a number of other reasons. Take-up rates of prescriptions have a social gradient due to economic constraints for poorer families to finance medication consistently, and at-risk families may be less inclined to follow medical advice or to comply with medical treatment despite negative side effects. Studies find that this applies for health care in general [50–51] and for mental health care and ADHD treatment in specific [52–53]. Such social gradient patterns may also help explain the existence of APT patterns in data. However, for differences in background characteristics to be important confounding factors, background characteristics should both correlate with the allocation into treatment patterns *and* simultaneously have a direct effect on the GPA outcomes [54].

To evaluate the extent that omitted variables bias our results we estimate Eq (1) both with and without observed covariates. If the differences between CPT, APT, and DPT are unaffected by including observed covariates, this indicates either that observed background characteristics are uncorrelated with our treatment indicators or with our outcome of interest. If so, this may also suggest that the observed differences in outcomes across CPT, APT, and DPT are unaffected by unobserved covariates as well, which in turn makes a causal interpretation of the treatment estimates more likely.

During the last 20 years, the number of medical products available has increased, while existing medical products have been enhanced [55–56]. Such developments may increase the probability that individuals continue medical treatment for ADHD once they start. However, the observed changing rates of DPT, APT, and CPT across time may also reflect a changing composition of the students in our data (students diagnosed with ADHD who commence treatment) across births cohorts. This could lead to selection bias. To control for this, we use a two-stage Heckman selection estimator to correct for sample selection into our data across birth cohorts [57]. As exclusion restrictions, we use children’s residential region at age three and their birth cohort in the selection equation only [58]. The propensity to prescribe medication differs across Danish regions [15,59], wherefore region of residence at a young age likely affects the propensity to enter medical ADHD treatment. As medical treatment has become increasingly more widespread, birth cohort may also affect the propensity to enter medical treatment. Birth cohort does not affect GPA because grades are by construction independent across cohorts in accordance with the grading legislation of the Danish compulsory schools [60]. We control for region of residence at graduation in the main equation to allow for the effect of unobservables across regions.

To further test whether we can view treatment patterns as an exogenous indicator of nonresponse to medical treatment, we use the availability of a placebo treatment group: students, who commence medical treatment *after* graduation (thus after receiving their grades) and for whom we should expect no treatment effect on their GPA (see Johansen et al. [61] for a similar design). Estimating Eq (1) on the placebo sample should yield no effect unless nonresponders (DTP) are selected on unobservables compared to responders (CPT) and ambiguous responders (APT). Children in the placebo and the treatment sample do on average differ in the timing of their treatment, and this may imply additional underlying differences in their background characteristics, as Table 1 shows. However, as we show in the result section the differences across DPT, APT and CPT are statistically insignificant indicating that estimated effects are unaffected by the inclusion of covariates in the treatment sample. Hence, it is unlikely that the differences in estimated effects with and without controls are important for comparing the differences between DPT, APT and CPT across the treatment and placebo sample. Moreover, from our data we find that students in the treatment sample and placebo sample are equally negatively affected by having an ADHD diagnosis in terms of their academic performance. This speaks in favor of comparability of the treatment and the placebo sample.

## Results

In this section, we present main results estimating Eq (1) on the sample of children diagnosed with ADHD.

Panel A in Table 3 shows estimation results for standardized exam GPA, whereas panel B shows estimation results for standardized teacher evaluation GPA. We replicate all treatment sample estimations in the placebo sample for both outcomes. However, as the results for the placebo sample are almost identical we only show the more statistically efficient estimates with adjustment for selection.

**Table 3:** Estimation results for treatment variables and select control variables^a^. OLS regression models and Heckman two-stage sample selection model. Outcome is (A) Standardized Exam GPA and (B) Standardized Teacher Evaluation GPA. Treatment and placebo ADHD sample.

	(A) Standardized Exam GPA				(B) Standardized Teacher Evaluation GPA			
	Treatment sample			Placebo sample	Treatment sample			Placebo sample
	(1) OLS without controls	(2) OLS with controls^a^	(3) Two-stage with controls^a^^,^^b^	(4) OLS with controls^a^	(1) OLS without Controls	(2) OLS with controls^a^	(3) Two-stage with controls^a^^,^^b^	(4) OLS with controls^a^
DPT	-0.13	-0.22**	-0.22***	-0.07	-0.12	-0.18**	-0.19**	-0.07
	(0.07)	(0.07)	(0.07)	(0.04)	(0.07)	(0.07)	(0.07)	(0.04)
APT	-0.12**	-0.13**	-0.14**	-0.03	-0.09*	-0.11*	-0.12*	-0.01
	(0.05)	(0.05)	(0.05)	(0.03)	(0.05)	(0.05)	(0.05)	(0.03)
Male		-0.18***	-0.14**	-0.19***		-0.24***	-0.24***	-0.33***
		(0.04)	(0.06)	(0.03)		(0.04)	(0.06)	-0.33***
Immigrant		-0.10	-0.12	-0.28*		-0.04	-0.03	-0.21
		(0.16)	(0.16)	(0.13)		(0.16)	(0.16)	(0.12)
Descendant of immigrant		0.05	0.01	0.07		0.04	0.04	0.12
		(0.13)	(0.13)	(0.11)		(0.13)	(0.13)	(0.11)
Mother diagnosed with ADHD		0.16*	0.29	0.22***		0.20**	0.21	0.20***
		(0.06)	(0.16)	(0.05)		(0.06)	(0.16)	(0.05)
Father diagnosed with ADHD		0.04	0.15	0.15*		0.00	0.02	0.16*
		(0.08)	(0.15)	(0.08)		(0.08)	(0.15)	(0.08)
Mother’s education ISCED 2		-0.03	0.00	-0.06		0.02	0.03	-0.06
		(0.08)	(0.08)	(0.05)		(0.08)	(0.08)	(0.05)
Mother’s education ISCED 3A		0.60***	0.62***	0.52***		0.46***	0.46***	0.48***
		(0.09)	(0.09)	(0.07)		(0.09)	(0.09)	(0.07)
Mother’s education ISCED 3C		0.08	0.10	0.12*		0.09	0.10	0.12*
		(0.08)	(0.08)	(0.05)		(0.08)	(0.08)	(0.05)
Mother’s education ISCED 5B		0.31***	0.32***	0.43***		0.26**	0.26**	0.43***
		(0.09)	(0.09)	(0.06)		(0.09)	(0.09)	(0.06)
Mother’s education ISCED 5AC		0.71***	0.69***	0.66***		0.64***	0.63***	0.67***
		(0.13)	(0.13)	(0.10)		(0.13)	(0.13)	(0.10)
Father’s education ISCED 2		0.04	0.04	-0.07		-0.03	-0.03	-0.07
		(0.07)	(0.07)	(0.05)		(0.07)	(0.07)	(0.05)
Father’s education ISCED 3A		0.50***	0.49***	0.36***		0.37***	0.38***	0.34***
		(0.10)	(0.10)	(0.08)		(0.10)	(0.10)	(0.08)
Father’s education ISCED 3C		0.09	0.09	0.02		0.04	0.04	0.03
		(0.06)	(0.06)	(0.04)		(0.06)	(0.06)	(0.04)
Father’s education ISCED 5B		0.32***	0.31***	0.18**		0.29***	0.29***	0.17**
		(0.08)	(0.08)	(0.06)		(0.08)	(0.08)	(0.06)
Father’s education ISCED 5AC		0.37***	0.35***	0.48***		0.44***	0.44***	0.44***
		(0.10)	(0.10)	(0.08)		(0.10)	(0.10)	(0.08)
Father’s education ISCED 6		0.27	0.19	1.01**		0.45	0.41	1.08**
		(0.40)	(0.40)	(0.37)		(0.40)	(0.40)	(0.37)
Constant	-0.54***	1.90**	0.96	1.66**	-0.65***	1.53*	1.41	0.76
	(0.04)	(0.63)	(1.17)	(0.53)	(0.04)	(0.63)	(1.17)	(0.53)
Lambda			0.14				0.01	
			(0.15)				(0.15)	
Rho			0.16				0.02	
*R*^2^	0.00	0.15	-	0.19	0.00	0.13	-	0.19
Observations	2659	2659	567322	3785	2659	2659	567322	3785
Uncensored observations	-	-	2659	-	-		2659	-

*Source*: Own calculations on data from Statistics Denmark.

* *p<*0.05

** *p<*0.01

*** *p<*0.001.

Standard errors reported in parentheses. DPT: Discontinued Pharmacological Treatment, APT: Ambiguous Pharmacological Treatment, CPT: Continuous Pharmacological Treatment (reference category). GPA: Grade point average. Outcome variables are standardized within the total population sample. Treatment sample: Children who begin medical ADHD treatment *prior to* compulsory school graduation. Placebo sample: Children who begin medical ADHD treatment *after* compulsory school graduation.

^a^ Models with controls also include parent age at childbirth, unemployment rate, income, negative income, as well as individual level information on birth weight, gestational age, age at treatment start, and being graded with the new grading scale. Dummies for missing information on all relevant variables are also included. The two-stage outcome model also includes residential region at graduation age. Full model estimates are available upon request.

^b^ Results from the Heckman two-stage selection model estimates of birth cohort and residential region at age three available upon request.

Model 1 in panel A reports differences across treatment patterns, such that children who follow continuous treatment (CPT) have significantly higher exam GPAs compared to those, who discontinue treatment partly (APT). The effect of DPT (full discontinuation) is -0.13, yet insignificant, and the effect of APT is -0.12 and significant. Including control variables (model 2) further increases these effects to -0.22 for DPT and -0.13 for APT, albeit estimates with controls are not significantly different from the estimates without controls. Nevertheless, controlling for background characteristics we thus find more than a fifth standard deviation difference between DPT and CPT and more than a tenth of a standard deviation difference between APT and CPT. Controlling for selection into our sample of initially treated students (model 3) has virtually no impact on the estimated effects across treatment groups, hence sample heterogeneity across birth cohorts seems negligible.

For the placebo sample (model 4), we find no differences across treatment groups, indicating no selection bias on unobservables for the placebo sample into DPT, APT and CPT. If we are willing to assume that this also applies for the treatment sample—supported by the fact that including controls did not change parameters in the treatment sample—the results support a causal interpretation of the effect of medication on GPA. One may argue that the placebo sample is not entirely comparable with the treatment sample, as they are diagnosed a considerable time after their exams. However, restricting the placebo sample to a narrower age range (only including students beginning treatment no later than age 24 or students beginning treatment no later than five years from graduation) does not alter our conclusions.

Inspecting the effect of the covariates on the outcome, we find much the expected sizes and signs: Males have lower exam GPA. Parent educational attainment level is positively correlated with exam GPA. Children whose mother is diagnosed with ADHD have a slightly higher GPA. The reason for mothers (and partly fathers) diagnosis being positively associated with GPA could be the fact that we study a selected sample; those diagnosed with ADHD (irrespective of their subsequent treatment pattern in terms of DPT, APT or CPT). Within this sample, parental experience with a diagnosis may prove to be beneficial. For the total population, however, the association is reversed; on average, students, whose parents have ADHD, obtain lower grades than students, whose parents do not have an ADHD diagnosis.

When re-estimating regressions including an interaction between duration and treatment pattern, we find that duration (measured in months) in itself has a very small yet significant positive influence on our outcome, but the interactions are nonsignificant. Thus, we do not find evidence that our main effects of treatment patterns vary with duration.

Comparing the covariate estimates for the treatment and placebo sample reveals similar sizes of the estimates for most control variables. This indicates that the selection process into the two samples is similar. Had they been different, we should have seen marked differences between the estimates because different selection processes would have yielded different selection bias in the estimated coefficients.

Panel B in Table 3 presents results for standardized teacher evaluated GPA. Treatment estimates are of similar magnitude compared to the estimates for exam GPA, i.e. .18 for DPT and .11 for APT (model 2). In addition, the control variables have similar effects on both GPA outcomes.

In sum, we find evidence of a positive effect of medical treatment on school-leaving exam and teacher evaluation GPAs for students diagnosed with ADHD, who enter treatment. Our results are robust to the inclusion of control variables and selection into the group of treated students. Moreover, there are no differences in GPA outcomes across treatment patterns for students beginning treatment after receiving their school-leaving GPA, which support our main results.

## Conclusion

In this study, we find that increased efficiency of medical treatment of ADHD can alleviate a substantial part of the GPA gap between children diagnosed with ADHD and their peers. Our empirical analyses show substantial and significant negative effects from discontinued pharmacological treatment (DPT) and ambiguous pharmacological treatment (APT) on school-leaving teacher evaluation and exam GPAs compared to consistent treatment (CPT). Treatment effects are synonymously negative, in the sense that both DPT and APT lower GPA across various model estimations and are robust to the statistical control for a large set of covariates and selection models.

Teacher evaluation GPA likely reflects both academic performance and student behavior in class. In this respect, teacher evaluated GPA reflects non-cognitive skills over and above the cognitive skills reflected in exam GPA. Finding that treatment effects are similar for teacher assessed performance (teacher evaluation GPA) and exam performance (exam GPA) supports the notion that medical treatment raises student academic performance and not merely alters student behavior in class.

Our main findings support an overall beneficial effect of medical treatment on long-term individual academic performance of children diagnosed with ADHD. Relying on the fact that national guidelines only recommend medical ADHD treatment for moderate to severe cases, children in the sample likely have moderate to severe symptoms, in which case benefits may exceed negative side-effects of treatment. In addition, Danish treatment rates of ADHD are more than 80 percent lower than, for example, US treatment rates [15]. Although due consideration should be given to the necessity of medicine versus alternative modes of interventions or combined treatment, medication may help certain children diagnosed with ADHD to function in an everyday school context when baseline-treatment levels are of a moderate size.

As medical treatment affects school-leaving GPA of individuals diagnosed with ADHD, treatment likely influences subsequent educational and vocational tracks as well; for example choice of post-secondary tracks, dropout rates, unemployment propensity, adult profession, and adult income as these are all strongly correlated with school-leaving GPA. International studies have also found evidence of such effects from ADHD diagnosis. However, due to interdisciplinary gaps between social and medical studies, research is needed that can adequately account for diagnosis *and* treatment as well as for the endogeneity when studying long-term social outcomes of children diagnosed with ADHD.

### Limitations

This study finds that medicine exerts a profound influence on the educational outcomes of children diagnosed with ADHD. Methodologically, our analysis rests on the stable unit treatment value assumption, implying that potential individual outcomes are unrelated to the treatment status, outcomes or characteristics of other individuals. However, children with ADHD are part of social contexts—school classes, families, etc.—and the individual effects of treatment presented should ideally be construed in its context. Therefore, additional research should study the impact of ADHD treatment on peers and families. A few recent studies suggest that ADHD and medical treatment hereof affects academic outcomes of non-ADHD siblings [8] and classroom peers [62]. Further, our effects are found in a context where only severe or moderate cases of ADHD are recommended to initiate pharmacological treatment. Hence, further studies are needed to assess the effects of treatment on long-term academic outcomes in contexts where less severe cases are also treated.

### Perspectives

More than four decades of sociological research has voiced concerns about medicalization, social control, and the social construction of problem behaviors [16–18]. Recent research shows that increasing medical treatment rates of ADHD in populations with high baseline-treatment levels likely has adverse consequences for children [63]. Yet, it remains a well-documented fact that children with behavioral and mental health problems, such as ADHD, have poorer later life outcomes than their peers. In addition, research from countries with low baseline-levels of ADHD treatment consistently shows that in this context, medical treatment lowers problem behavior [59]. Our study adds to this research and our results indicate that medical treatment can improve the educational trajectories of children diagnosed with moderate to severe symptoms ADHD. Furthermore, in our theory section we outline two distinct but interrelated pathways through which ADHD likely affects educational outcomes—a direct, neurobiological impact on cognition and attention, as well as an indirect pathway changing learning environments. Further knowledge on the importance of each of these pathways in shaping children’s educational outcome could provide valuable insight into how to best address the educational challenges faced by children with behavioral problems, and should be the subject of future research.
